# Sonoelastography of the Shoulder: A Narrative Review

**DOI:** 10.3389/fresc.2021.704725

**Published:** 2021-07-06

**Authors:** Arash Babaei-Ghazani, Carl-Elie Majdalani, Dien Hung Luong, Antony Bertrand-Grenier, Stéphane Sobczak

**Affiliations:** ^1^Department of Physical Medicine and Rehabilitation, Neuromusculoskeletal Research Center, Iran University of Medical Sciences, Tehran, Iran; ^2^Department of Physical Medicine and Rehabilitation, Centre Hospitalier de l'Université de Montréal, Montréal, QC, Canada; ^3^Département de chimie, biochimie et physique, Université du Québec à Trois-Rivières, Trois-Rivières, QC, Canada; ^4^Département d'anatomie, Université du Québec à Trois-Rivières, Trois-Rivières, QC, Canada; ^5^Groupe de recherche sur les affections neuromusculosquelettiques (GRAN), Université du Québec à Trois-Rivières, Trois-Rivières, QC, Canada; ^6^Chaire de recherche en anatomie fonctionnelle, Université du Québec à Trois-Rivières, Trois-Rivières, QC, Canada

**Keywords:** sonoelastography, strain elastography, shear wave elastography, mechanical properties, shoulder, rotator cuff, tendinopathy, ultrasound

## Abstract

Sonoelastography is a relatively new non-invasive imaging tool to assess the *in vivo* qualitative and quantitative biomechanical properties of various tissues. Two types of sonoelastography (SE) are commonly explored: strain and shear wave. Sonoelastography can be used in multiple medical subspecialties to assess pathological tissular changes by obtaining mechanical properties, shear wave speed, and strain ratio data. Although there are various radiological imaging methods, such as MRI or CT scan, to assess musculoskeletal structures (muscles, tendons, joint capsules), SE is more accessible since this approach is of low cost and does not involve radiation. As of 2018, SE has garnered promising data in multiple studies. Preliminary clinico-radiological correlations have been established to bridge tissue biomechanical findings with their respective clinical pathologies. Specifically, concerning the shoulder complex, recent findings have described mechanical tissue changes in shoulder capsulitis. The long head of the biceps and supraspinatus SE were among the recently studied structures with conditions regarding impingement, tendinosis, and tears. Since ultrasonography has established itself as an important tool in shoulder evaluation, it completes the history and physical examination skills of the clinicians. This study will provide an update on the most recent findings on SE of shoulder structures.

## Introduction

**Figure 1:**
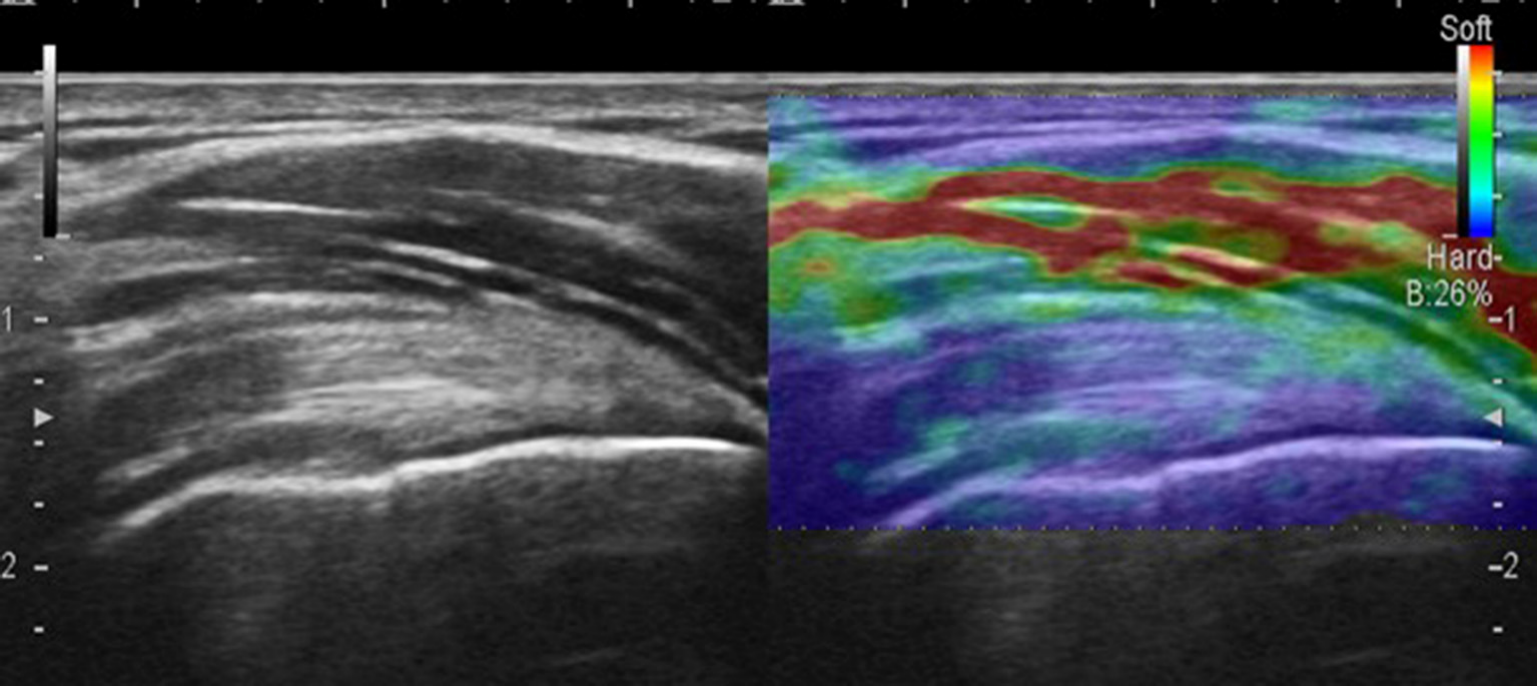
Strain sonoelastography of the supraspinatus tendon.

**Figure 2:**
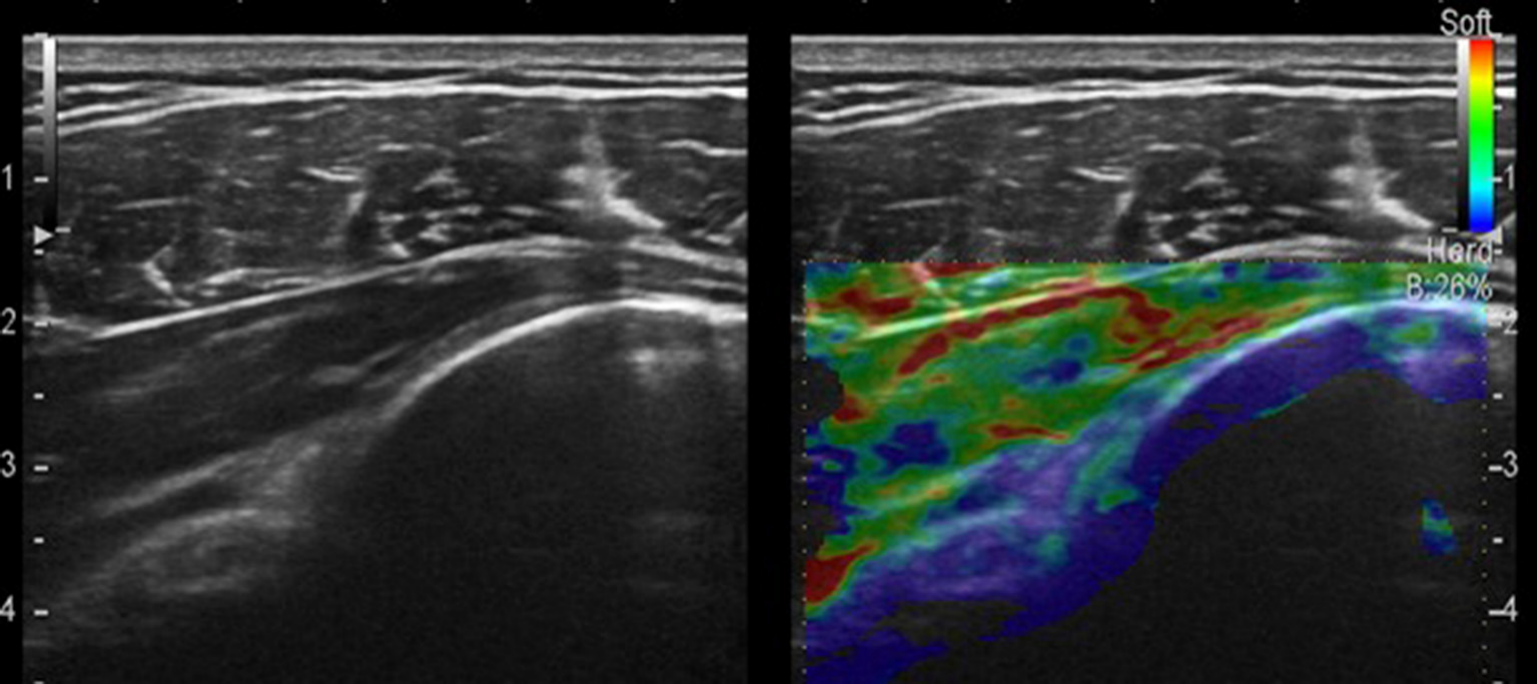
Strain sonoelastography of the infraspinatus tendon and posterior capsule.

Sonoelastography is a relatively new and non-invasive ultrasound (US) technique that provides information about mechanical properties of tissues, such as stiffness, based on the palpation method (1). There are two main types of elastography: static (strain) and dynamic (shear waves). In strain sonoelastography (SSE), echo signals before and after compressions are measured to calculate the strain. SE offers only qualitative data following external pressure tissue loading (Figures 1, 2). Dynamic SE (SWE) uses an acoustic radiation force to create and propagate shear waves. This method measures the shear wave speed to evaluate mechanical properties of tissues to visualize tissue displacements to allow the examiner to infer information regarding the tissular biomechanics (1–3). Both methods are operator-dependent and have yielded favorable inter-rater reliability (4, 5). This narrative review serves as an update on the novel literature concerning the usage of SE of the shoulder since 2018 until to date.

## Methodology

A thorough search of the most recent literature was conducted. A systematic search of MEDLINE (PubMed), EMBASE (Ovid), and Web of Science was performed to identify relevant publications from January 2018 to May 2021. The following text words, Medical Subject Headings (MeSH) terms, and Boolean operators were used: “elastography or sonoelastography or elastosonography” and “shoulder,” “shoulder joint,” “shoulder pain” “biceps,” “capsulitis,” “labrum,” “deltoid,” “infraspinatus,” “supraspinatus,” “rotator cuff,” “teres major or minor,” “acromioclavicular joint,” “scapula,” “coracohumeral,” “coracoacromial,” “coracoclavicular,” “quadrangular space,” “triangular space or interval,” and “spinoglenoid.” We excluded all articles not related to shoulder region or elastography or SE. The search was conducted with English and French language restriction. A further triage was performed using the COVIDENCE sorting platform to retain the final articles reviewed in this article.

## Results

In recent years, several articles were published for sonoelastographic evaluation of different shoulder pathologies. A systematic review by Chiu et al. analyzed 11 studies covering the use of SE in pathologies such as adhesive capsulitis, rotator cuff tendinopathy, and tear (targeting mainly the supraspinatus tendon as well as the infraspinatus tendon and deltoid muscle) (6). They found that, in adhesive capsulitis, both supraspinatus and infraspinatus tendons showed a decrease in elasticity. Moreover, there existed no significant differences in biomechanical properties of rotator cuff tendons presenting with tendinopathy and/or tears when using both SE and SWE.

Demirel et al. explored the diagnostic value of supraspinatus muscle SE in supraspinatus impingement syndrome in a cross-sectional study (7). Measurements were made on 41 patients with unilateral impingements to compare the results with the normal side. They observed a lower strain ratio value in the supraspinatus muscle on the impingement syndrome side. This finding infers that a low strain ratio indicates high elasticity. They reported a cut-off value of 0.495 (strain ratio > 0.495) with sensitivity and specificity of 75.6 and 78%, respectively. They concluded that strain ratio measurement with SE can be a practical, non-invasive, and inexpensive diagnostic tool for shoulder impingement disease.

Mackintosh et al. used 2D shear wave SE to predict fatty infiltration of the supraspinatus muscle as a prognostic factor in determining supraspinatus tendon repair failure (8). They evaluated the anterior and posterior portions of the superficial half of the supraspinatus muscle in 152 shoulders. They compared them to MRI Goutallier grading of fatty infiltration. Only one participant had high-grade fatty infiltration (grade 3 or 4) in their study. They concluded that random SWE sampling throughout the superficial supraspinatus muscle highly correlates with MRI grading but lacks accuracy. Moreover, the presented method did not show that 2D SWE can replace traditional MRI grading. Finally, no additional information on the severity of the tears was offered.

Brage et al. explored the discriminative validity of ultrasound SE for patients with painful supraspinatus tendinopathy (9). They compared 30 patients with supraspinatus tendinopathy with 30 healthy controls for the associations between SE and MRI, ultrasonographic tendon thickness, and the Disabilities of the Arm, Shoulder, and Hand (DASH) questionnaire. They reported that patients with supraspinatus tendinopathy may have had a softer supraspinatus tendon than healthy control shoulders. No correlation was found between SSE values and tendon thickness. Tendon softness can be due to histological loss of collagen tissue and fatty infiltration.

Brage et al. assessed the intra-rater and inter-rater reliability of SE in the supraspinatus tendon evaluation (4). They included 20 participants with MRI confirmed supraspinatus tendinosis and 20 asymptomatic volunteers to measure raw values and strain ratios in three supraspinatus tendon areas. They obtained excellent intra-rater and inter-rater reliability for raw values and ratios using the deltoid muscle as a reference and good results for ratios using gel pads as reference tissue (Table 1). The reliability was substantial-to-almost perfect for the color scale ratings and fair-to-almost perfect for the number of red/yellow color lesions. They highlighted that the SE is highly operator-dependent, depending on the pressure applied. This may affect the external validity of SE in clinical settings. They mentioned different factors such as manual compression, reference tissues, and operator train level as limitations of this technique.

**Table 1:** Summary of the reviewed articles.

**Article**	**Correlation**	**Area examined**	**# Subjects (F:M) age (range)**	**Type of EU**	**Control group**	**Result**
Chiu et al. (6)	Nil	SS, IS, DEL	Nil	SWE + SE	Nil	Reduced elasticity in SS and IS tendons in adhesive capsulitis No differences in tendon elasticity in rotator cuff pathologies (tears and tendinopathy).
Demirel et al. (7)	MRI	SS	41 subjects (18:23) 53.1 (38–70)	SWE	No	Lower strain ratio value in the supraspinatus muscle on the impingement syndrome side (Negative, 0.74; Positive 0.31). Positive cut-off value of 0.495 (strain ratio > 0.495) with sensitivity and specificity of 75.6 and 78%, respectively.
Mackintosh et al. (8)	MRI	SS	152 shoulders (48:101) 54 (32–83)	SWE	No	They concluded that random SWE sampling throughout the superficial supraspinatus muscle highly correlates with MRI grading but lacks accuracy.
Brage et al. (9)	MRI	SS	60 subjects 30 pathological (17:13); 30 healthy (21:9) Age: 51; 47	SWE + SE	Yes	They reported that patients may have had a softer supraspinatus tendon than healthy control shoulders. No correlation was found between strain sonoelastography values and tendon thickness.
Brage et al. (4)	MRI	SS	40 subjects 20 (14:6) pathological (MRI confirmed); 47.8 y.o 20 (11:9) healthy (no MRI) 25.7 y.o	SE	Yes	They obtained excellent intra-rater and inter-rater reliability for raw values and ratios using the deltoid muscle as reference LWk: 0.89 and 0.78 respectively), and good results for ratios when using gel pads as reference tissue (LWk: 0.73 and 0.70, respectively). For the colour scale (performed without gel), the intra-rater and inter-reater reliability were relatively similar. (LWk 0.76 to 0.79 and 0.71 to 0.81, respectively). For the number of lesions, intra-rater and inter-rater reliability faired better in the medial and middle part of the tendon (LWk:0.82 and 0.75 and 0.67 and 0.63, respectively) than in the lateral portion (LWk: 0.40 and 0.24, respectively).
Vasishta et al. (10)	MRI	SS	25 subjetcts (7:18) 41.7 y.o	SE	No	They reported a good correlation between the supraspinatus tendon strain ratios and the MRI grade: the strain ratio decreased with increasing severity of tendinopathy. Perfect negative correlation was found (*r* = −0.058; *p* = 0.0094) between MRI grade and strain ratio
Yoo et al. (11)	MRI, CT	SS	54 subjects 27 partial thickness; 27 full thickness No details on age	SWE	No	They did not find any statistically significant difference in elasticity values between normal (Median elasticity 94.65 kPa) and torn supraspinatus tendons or between partial-thickness tears (Median elasticity 96.83 kPa) and full-thickness tears. (Median elasticity 93.80 kPa). No statistically significant difference. SWE values were significantly higher in chronic supraspinatus tendon tears symptomatic for more than one year (median elasticity 105.10 kPa for the more than 1 year group vs. 92.20 kPa for the less than 1 year group)
Fontenelle et al. (2)	None	SS	38 healthy subjects (age 20-35) vs. 18 older adults (over 60 y.o)	SWE	Yes	A statistically significant difference was observed in their study between the two age groups and SWE modulus showed a significant decrease in the over 60 years-old age group (median shear modulus of 17.92 kPa vs. 23.28 kPa in younger adults) *p* = 0.033
Hackett et al. (5)	None	SS	20 subjects (14:6) Normal group: 25 y.o (21–53) Tendinopathic group 49 (27–69)	SWE	Yes	SWE showed less stiffness in tendinopathic than normal supraspinatus tendons. There was moderate inter-rater reliability (ICC = 0.45) There was excellent intra-rater reliability (ICC = 0.96)
Zhou et al. (3)	None	SS	87 subjects (41:46) 51.3 y.o Control group: 30 volunteers (15:15)	SWE	Yes	Average SWE stiffness value of patients with supraspinatus tendinopathy (60.6 kPa ± 11.5) was greater than healthy subjects (26.12 kPa ± 4) *p* = 0.001 YM positively correlated to VAS (*r* = 0.564; *p* < 0.001) YM negatively correlated with Constant Murley Score (CMS) (*r* = −0.411; *p* < 0.001)
Nocera et al. (12)	MRI	SS	12 subjects (5:7) 61 y.o (44-72) with full thickness tear, all were post of for RC repair	SWE	No	Although not statistically significant, postoperative SWE initially decreased and later increased. Tendon-Muscle Ratio (TMR) followed an opposite trend at 3 and 6 months.
Kim et al. (13)	None	DEL, SS, IS	12 male healthy volunteers 30 y.o	SWE	No	They concluded that shoulder muscle activity can be measured with ultrasound SWE in both static and dynamic modes Intra-rater and inter-rater reliability (as per the intraclass correlation (ICC) was excellent in all examined planes. For intra-rater reliability, in the longitudinal plane, ICC yielded 0.96, 0.97, 0.96 for abduction, external rotation, and scaption respectively. For intra-rater reliability, in the transverse plane, ICC yielded 0.90, 0.94, 0.91 respectively for the aforementioned movements. For inter-rater reliability, in the longitudinal plane, ICC 0.97, 0.91, 0.93 respectively for the aforementioned movements. For inter-rater reliability, in the transverse plane, ICC yielded 0.87, 0.90, 0.85 respectively for the aforementioned movements.
Sahan et al. (1)	MRI	LHB	20 subjects with LHBT (10:10) 54.5 y.o ± 10.1 20 healthy subjects (10:10) 47.5 ± 11.9	SWE	Yes	Statistically significant difference in terms of elasticity patterns between the tendinosis (transverse plane: 38.3 kPa; longitudinal plane 39.4 kPa) and normal group (transverse plane 18.6 kPa; longitudinal plane 20.6 kPa) *p* < 0.001
Yun et al. (14)	None	SS, IST	20 pathological subjects (14:6) 53.5 y.o ± 7.9 18 healthy subjects (6:12) 52.6 ± 10.5	SWE + SE	Yes	Both velocity and stiffness in SWE were higher, and the strain ratio in SE Lower in participants with symptomatic shoulders than in those with normal shoulders (*p* < 0.001)
Wada et al. (15)	None	SSt, SS, IS, TMi, UT, LT, CHL, LHB, GHPC	32 subjects with frozen shoulder (19:13); 59.4 y.o 15 in the freezing phase (5:9); 54.9 y.o 17 in the frozen phase (9:8); 63.4	SWE	No	The SWE values for the SSp and ISp tendons in the freezing phase and the CHL in the frozen phase were significantly greater on the affected side than the unaffected side (mean ± SD, 280.4 ± 125.3 versus 178.1 ± 73.3, 318.4 ± 110.7 versus 240.8 ± 91.5, and 287.2 ± 135.3 versus 214.1 ± 91.1 kPa, respectively; *P* < 0.05). The posterior capsule in both the freezing and frozen phases and the CHL in the frozen phase were significantly thicker on the affected side than the unaffected side (1.3 ± 0.2 versus 0.9 ± 0.3, 1.2 ± 0.4 versus 0.9 ± 0.3, and 4.4 ± 1.4 vs. 3.3 ± 1.1 mm; *P* < 0.01).
Lin et al. (16)	MRI	SS	88 subjects (46:42) 55 ± 15 y.o	SWE	No	SWE can detect biomechanical changes in the supraspinatus muscle that are not morphologically evident on gray-scale US. Statistically significant differences when comparing tendon grade 3 with grades 1, 2, and 4 (*p* = 0.018, 0.025, 0.014), respectively
Itoigawa et al. (17)	MRI	SS	60 subjects (25:35) with SS tear 66.1 y.o (45–80)	SWE	No	SWE value of the repaired tendon had decreased significantly from its 1-week value by 3 months after ARCR in the both Partial-Small and Medium-Large groups. Values of SWE depended on the size of the tear. Increased SWE values at 1 month after arthroscopic RC repair may predict recurrent tears after surgery
Itoigawa et al. (18)	MRI	SS	38 subjects with full SS tear (15:23) 66.7 y.o (46–81)	SWE	No	Highest correlation with the supraspinatus musculotendinous stiffness was with the SWE modulus of the posterior deep region of the muscle (*r* = 0.69; *p* < 0.01) Correlation between supraspinatus tear SWE evaluation and MRI Goutallier staging was statistically significant (*r* = 0.49; *p* < 0.01).
Hsu et al. (19)	None	SS, LHB	60 subjects with subacromial impingement syndrome (27:33) for a total of 120 shoulders 58.3 y.o ± 2.1	SE	Yes	No differences in tendon elasticity as evaluated by SE at 3 months post corticosteroid injectate in SS and LHBT's mechanical properties.

*SS, suprastinatus; DEL, deltoid; IS, infraspinatus; LHB, long head of biceps brachii; CHL, coracohumeral ligament; TMi, teres minor; UT, upper trapezius; LT, lower trapezius; GHPC, glenohumeral posterior capsule; SWE, shear wave elastography; SE, strain elastography; LWk, linear weighted Cohen's kappa; ICC, intraclass coefficient coefficients*.

Itoigawa et al. investigated changes in the supraspinatus muscle and tendon stiffness after arthroscopic rotator cuff (RC) repair (17). They compared the SWE in patients with recurrent tears to patients with healed RC. They evaluated 60 patients with supraspinatus tears requiring arthroscopic RC repair with serial SWE. Evaluations were done once before surgery and postoperatively at 1 week with monthly follow-ups for 6 months. They reported significantly greater SWE value in repaired tendons at 1 week after surgery than at 3 and 6 months. SWE value for the supraspinatus muscle at 1 month after surgery in the healed group was lower than pre-surgery and 4, 5, and 6 months after surgery. It was also lower than that at 1 month after surgery in the re-tear group. No significant differences between time points in the supraspinatus muscle SWE values in the re-tear group were found. The SWE value of the muscle in the re-tear group was greater than in the healed group at 1 month post-operation. They concluded that increased SWE values at 1 month after arthroscopic RC repair may predict recurrent tears after surgery rather than evaluating the tendon.

Lin et al. aimed to determine whether SWE can detect biomechanical changes in the supraspinatus muscle concerning supraspinatus tendon abnormality before US grayscale changes (16). They evaluated 110 shoulders of 88 patients from 2013 to 2018 using a grading score in order of increasing supraspinatus tendinosis/tear (1–4 scale) and increasing fatty infiltration (0–3 scale) probe in a longitudinal orientation. They reported no significant correlation between supraspinatus muscle or tendon grading and SWE. In the morphologically normal muscle grayscale US subgroup, there were significant differences in supraspinatus muscle SWE in tendon grade 3 compared with other grades (increased muscle SWE in grade 3). They concluded that SWE can detect biomechanical changes in the supraspinatus muscle that are not morphologically evident on the grayscale US, especially supraspinatus tendon partial tears with moderate to severe tendinosis.

Vasishta et al. assessed the relationship between tendon stiffness on SE and supraspinatus tendinopathy grading on MRI (Grade I, normal; Grade II, mild tendinopathy; Grade III, moderate tendinopathy; and Grade IV, marked tendinopathy) in 25 patients (10). They reported a good correlation between the supraspinatus tendon strain ratios and the MRI grade: the strain ratio decreased with increasing severity of tendinopathy. Sonoelastography might help to predict improvement or worsening of supraspinatus tendinopathy. They acknowledged the possibility of SE value in the supraspinatus tendinopathy rehabilitation.

Yoo et al. investigated the value of SWE for the estimation of the supraspinatus tendon tear chronicity (11). They evaluated 54 shoulders with supraspinatus tendon tear (27 with a partial-thickness tear and 27 with a full-thickness tear). They did not find any statistically significant difference in elasticity values between normal and torn supraspinatus tendons or between partial-thickness tears and full-thickness tears. However, they found a statistically significant difference in SWE concerning the duration of symptoms between 1 year or less versus longer than 1 year. They concluded that SWE values were significantly higher in chronic supraspinatus tendon tears symptomatic for more than 1 year. They suggested further studies with larger samples to determine SWE values as a surrogate marker of the chronicity in RC tendon tear before surgery.

Fontenelle et al. compared the mechanical properties of the supraspinatus tendon in two different age groups using SWE (2). They evaluated the right shoulder of 38 healthy individuals between 20 and 35 years old and over 60 years of age in a level of evidence III study. A statistically significant difference was observed in their study between the two age groups, and SWE modulus showed a significant decrease in the over 60 years old age group.

Hackett et al. evaluated the reliability of SWE to evaluate the stiffness of normal and tendinopathic supraspinatus tendons (5). They assessed inter- and intra-rater reliability for performing SWE by three raters with different experiences in the conventional US and obtaining three readings in three different examinations per subject over a 1-week period. They investigated 10 normal and 10 subjects with tendinopathic supraspinatus. They concluded that SWE showed less stiffness in tendinopathic than normal supraspinatus tendons. It was a reliable imaging technique to assess this tendon, especially when a single experienced musculoskeletal sonographer performed.

Zhou et al. explored the value of SWE in the treatment efficacy and prognostic evaluation of supraspinatus tendinopathy (3). Supraspinatus treatment ranged from non-pharmacological management to local injection based on the reported pain visual analog scale (VAS) of the subject. They assessed 87 patients with supraspinatus tendinopathy and 30 healthy volunteers. VAS and Constant-Murley Score (CMS) were used at different treatment courses with 1-year follow-up. They reported that the average SWE value of patients with supraspinatus tendinopathy was greater than healthy subjects (stiffness of the pathological tendon was greater than healthy tendon). It positively correlated with VAS and negatively correlated with its CMS. They concluded that SWE can objectively indicate the severity of supraspinatus tendinopathy.

Nocera et al. determined the healing response after RC repairs using a multimodality imaging approach with MRI, power Doppler and SWE (12). They evaluated 12 patients with unilateral, full-thickness, supraspinatus tendon tear in a cohort study with preoperative and postoperative (at 3 and 6 months post-operation) US and MRI. The MRI signal intensity ratio of tendon-to-deltoid muscle and vascularity by power Doppler and SWE were measured in repaired and asymptomatic control shoulders. Tendon-to-deltoid muscle ratio and vascularity of the tendon after repair initially increased and then decreased. Although not statistically significant, postoperative SWE initially decreased and later increased. They concluded that, in the RC repair, a temporal relationship existed between the MRI and US parameters with healing phases of the RC surgery.

Itoigawa et al. determined the feasibility of SWE to evaluate the RC muscle stiffness before arthroscopic RC repair with the intention to explore the surgical procedure's difficulty and compare SWE with the Goutallier stage on MRI (18). They investigated 38 patients with a full-thickness supraspinatus tear undergoing arthroscopic RC repair divided into two groups: >50% of the footprint was covered during the stiffness measurement or the incomplete with <50% footprint coverage. The Goutallier MRI stage of fatty infiltration and the SWE modulus were measured before surgery in posterior superficial, posterior deep, anterior superficial, and deep anterior regions of the supraspinatus muscle. They concluded that the highest correlation with the supraspinatus musculotendinous stiffness was with the SWE modulus of the deep posterior region of the muscle. They also mentioned that the SWE can predict the stiffness of the supraspinatus musculotendinous unit, and SWE may be used for pre-surgical planning.

Kim et al. evaluated the activity of the deltoid, supraspinatus, and infraspinatus muscles using SWE compared with the isokinetic dynamometry and surface electromyography methods on 12 volunteers (13). They found a linear relationship between SWE and isokinetic dynamometry in longitudinal and transverse ultrasonography planes of the muscles mentioned above. They had similar findings concerning surface electromyography. They reported excellent intra-observer and inter-observer reliabilities in all positions (abduction, external rotation, and scaption) with different ultrasonography planes (Table 1). They concluded that shoulder muscle activity can be measured with ultrasound SWE in both static and dynamic modes. It may be a useful tool to evaluate RC muscle activity relatively simply for changes in tissue tightness in shoulder disorders with increased soft tissue stiffness (i.e., adhesive capsulitis) or in pre-operative and postoperative conditions.

Yun et al. compared the elasticity of the supraspinatus and infraspinatus tendon in idiopathic adhesive capsulitis patients with a control group to evaluate the tendon elasticity relationship (14). They observed 25 shoulders with adhesive capsulitis and 24 normal shoulders. Both SWE and SE were performed in the oblique coronal plane at the neutral shoulder position for supraspinatus and infraspinatus tendons near the insertion to the greater tuberosity. Data for speed and stiffness from the SWE and strain ratio (subcutaneous fat/target tendon) from the SE of the supraspinatus and infraspinatus tendons were evaluated. They reported higher velocity and stiffness in SWE and lower strain ratio in SE in patients with symptomatic adhesive capsulitis than normal shoulders. They concluded that, in SWE and SE, supraspinatus and infraspinatus tendons were stiffer in adhesive capsulitis patients than in normal shoulders regardless of aging.

Sahan et al. investigated SE and SWE characteristics of the long head of the biceps tendon (LHBT) tendinosis compared with MRI findings (1). They evaluated 20 patients with an MRI diagnosis of tendinosis and 20 healthy individuals. They reported a statistically significant difference in elasticity patterns between the tendinosis and normal group (harder tissue in the tendinosis group) with very high sensitivity and specificity for the cut-off value of transverse 25.8 kPa and longitudinal, 24.6 kPa in SWE for tendinosis. They concluded that SE and SWE may be useful diagnostic tools for LHBT tendinosis than MRI concerning usability, cost-effectiveness, and patient preference.

Wada et al. used SWE to evaluate the stiffness of the capsule, RC tendons and muscles, the coracohumeral ligament (CHL), and LHBT in patients with frozen shoulder (15). They compared 32 patients with frozen shoulder, divided them into freezing and frozen phases, without RC tears to the unaffected shoulders. Patients with limited active and passive shoulder ROM (<100° of anterior flexion, <10° external rotation, and <L5 of internal rotation) were placed in the freezing phase if they reported severe pain (≥6/10 on VAS) while the frozen phase patients complained of moderate (≤5/10 on VAS) pain. They reported significantly greater SWE values for the supraspinatus and infraspinatus tendons in the freezing phase (phase I) and the CHL in the frozen phase (phase II) on the affected side. In the B-mode US, they observed a thicker posterior capsule in both the freezing and frozen phases and a thicker CHL in the frozen phase on the affected side compared with the unaffected side. They highlighted the added value of the changes in stiffness of the RC in SWE to the changes in the thickness of the capsule in the B-mode US for patients with frozen shoulder. They performed SWE with the transducer parallel to the fiber orientation for all evaluated tissues secondary to the high reliability of repeatability at this orientation.

Hsu et al. used SE to investigate whether corticosteroid injections influenced the elasticity of tendons (19). Considering the potentially deleterious effect of corticosteroids on the biomechanical properties of the tendon (i.e., tensile strength) has been reported in animal models, few studies investigated such impact on a human shoulder. Subjects with a subacromial impingement diagnosis were randomized into two groups. They would either receive an injection in the bursal side of the supraspinatus tendon or a dual-target injection, including the previously mentioned procedure to a peritendinous injection of the LHBT. The administered injectate was triamcinolone acetate at 40 mg (for the supraspinatus tendon) or 20 mg (for the LHBT). Follow-ups were scheduled at 1 and 3 months post-infiltration. The strain ratios of the studied tendons were similar at baseline when evaluated at the end of the follow-up period. They yielded no statistically significant differences and no subsequent rotator cuff tears. The investigators obtained the SE data reliably, as demonstrated by a pilot study where the intra-rater and inter-rater were obtained at 0.877 and 0.789, respectively.

## Conclusion

Possible barriers to the SE technique include operator dependency and limitations such as artifacts and reliability. During SE evaluation, it is important to mention the age, gender, muscle segment, shoulder position, and tension applied to the tendon or muscle of subject. In both SE and SWE methods, the biggest shortcoming seems to be choosing the region of interest (ROI) to be calculated with US machines by the Young modulus. For shear wave SE evaluation, there is controversy in previously published articles with regard to terminology. The shear wave SE does not directly measure stiffness, which is the resistance of a material to elastic deformity (20). It measures the shear wave speed through said material. The wave propagation speed is related to the stiffness of the tissue: the faster the shear wave travels, the stiffer the tissue. A specific limitation to this review lies within the research strategy methodology. Since this is a narrative review and not a systematic one, we narrowed our search to obtain only the latest pertinent publications.

Concerning recently published shoulder SE articles, shoulder capsulitis showed increased stiffness in supraspinatus and infraspinatus tendons and CHL on SWE evaluation (14). For fatty infiltration assessment and correlation with MRI grading, two recent studies with different conclusions and further studies seem necessary (5, 12). One study showed a softer supraspinatus tendon after 60 years of age. Another study showed increased stiffness in LHBT in tendinosis conditions (1, 2). In post-surgical status after supraspinatus tendon repair, SWE seems to show different short-term and long-term results concerning two newly published articles (8, 11). In previously published papers, there are controversies for SE changes (hardness vs. softness) of supraspinatus tendinopathic conditions. Finally, in one recent study, corticosteroid injectate did not modify or disrupt tendon elasticity at 3 months post-infiltration (15). More studies in the upcoming future seem necessary to further clarify SE application in the diagnostic shoulder ultrasonography. Moreover, future research should include various therapies such as shock wave, nerve blocks, and orthobiologics and their subsequent SE findings.

## Author Contributions

AB-Gh, AB-Gr, and C-EM wrote the manuscript. DL, AB-G, and SS amended and finalized the final proof of the manuscript. All authors contributed to the article and approved the submitted version.

## Conflict of Interest

The authors declare that the research was conducted in the absence of any commercial or financial relationships that could be construed as a potential conflict of interest.
